# The large scale antibacterial, antifungal and anti-phage efficiency of Petamcin-A: new multicomponent preparation for skin diseases treatment

**DOI:** 10.1186/s12941-015-0087-z

**Published:** 2015-05-18

**Authors:** Mikayel Ginovyan, Andranik Keryan, Inga Bazukyan, Petros Ghazaryan, Armen Trchounian

**Affiliations:** Department of Microbiology, Plants and Microbes Biotechnology, Faculty of Biology, Yerevan State University, Yerevan, Armenia; Department of Pharmaceutical Chemistry, Faculty of Chemistry, Yerevan State University, Yerevan, Armenia

**Keywords:** Acetic acid, Petamcin-A, Antimicrobial activity, Anti-phage activity, Biologically active compounds, Skin infectious disease

## Abstract

**Background:**

Human and animal skin diseases of bacterial, fungal and viral nature and their complications are widespread and globally cause a serious trouble. Their prevalence is increasing mainly due to drug resistance. Consequently, demand has increased for new effective antimicrobial drugs, which also should be less toxic, possess a wider spectrum of action and be economically more beneficial. The goal was to investigate antibacterial, antifungal and anti-phage activity of Petamcin-A-a new multicomponent preparation. It contains acetic acid and hexamethylenetetramine as main active antimicrobial components, as well as phosphatidylcholine, tocopheryl acetate and glycerol as excipients.

**Methods:**

Bacteriostatic activity and minimal inhibitory concentrations of the preparation against various test-organisms were determined by agar well diffusion assay. Antifungal activity was tested by agar dilution assay. To explore anti-phage activity double agar overlay plaque assay was used. Nystatin, chlorhexidine and acetic acid were used as control agents for comparative analysis. Statistical analysis was done with GraphPad Prism 5.03 or R 3.1.0 software.

**Results:**

The results showed a higher activity of Petamcin-A against all bacterial and fungal test strains compared with its components or control agents. The preparation was more effective against tested gram-positive bacteria than gram-negative ones. Petamcin-A expressed bactericidal activity against almost all test strains. In addition, the preparation demonstrated high activity against T4 phage of *Escherichia coli* C-T4 completely inhibiting its growth. 5-fold diluted Petamcin-A also exhibited considerable activity reducing phage concentration by 2.6 Log_10_.

**Conclusions:**

Petamcin-A has a high antimicrobial activity against all tested strains of bacteria, yeasts and moulds. The preparation also exhibited high anti-phage activity. Moreover, taking into account that Petamcin-A has no observable toxicity on skin and its components are not expensive, it can be advantageous for management of various skin medical conditions.

## Introduction

The prevalence of bacterial, fungal and viral skin infectious diseases and their complications are continuously increasing throughout the world. Because of the limited number of therapeutic methods this problem is one of the most urgent challenges in biology and medicine [1–3]. The frequency of these diseases is particularly higher in developing countries, mainly due to lower level of their healthcare systems as well as inability of many patients to receive expensive treatment for a long period of time [4]. Treatment difficulties of these diseases mainly are a consequence of acquired resistance development by microorganisms towards commonly used drugs [5–8]. Therefore, it is not a coincidence, that 21^th^ century is considered as post-antibiotic era in order to highlight importance of the problem [9]. Another serious challenge of the field is the acquisition of pathogenicity by microorganisms previously considered as non-pathogenic, which could happen due to various mutations, increasing number of immune-compromised patients, *etc.* [10]. Hence, the development of new, more efficient drugs is of utmost importance. On the other hand, it is essential to create economically more beneficial antimicrobials affordable for a population living in poor regions. Another criterion of new antimicrobials is a wide spectrum of action, considering the fact that many skin infections are caused by various microbial associations [11].

Both topical and systemic antimicrobial agents could be used for treatment of skin infections depending on various conditions (infection stage, size of infected area, type of pathogens, depth of infections, *etc.*). Both of them have their advantages and disadvantages [12, 13]. Particularly, topical antimicrobials help to avoid systemic toxicity and side effects as well as to decrease the possibility of resistance acquisition. Moreover, they allow to apply high concentration of antimicrobials directly on the zone of infection. The main disadvantage of topical agents is a difficulty to supply active agents to affected area, especially during deep skin infections [12, 13].

The object of this study was the multi-component liquid preparation Petamcin-A, which was developed and patented as topical antifungal agent [14]. Preparation contains acetic acid, hexamethylenetetramine, phosphatidylcholine, tocopheryl acetate and glycerol [14]. It has been successfully used during recent years for treatment of skin fungal diseases. This preparation does not have any noticeable toxicity and does not express any side effects [14, 15]. According to the preliminary data, under the influence of this preparation, statistically significant normalization of cytotoxic and membranolytic lysophosphatides and phospholipase A2 activity are observed at experimental animals [15]. This testifies about Petamin-A’s membrane stabilizing properties. The preparation also expresses antioxidant activity which is partially connected with its phosphatidylcholine and tocopheryl acetate components [14]. As it was shown, the preparation promotes fast purification of the leukocytic and necrotic mass of the wound, suppresses the activity of inflammatory reactions, stimulates cellular proliferation, and promotes the growth of connective tissue and regeneration of wound epithelium [15].

Two components of Petamcin-A-acetic acid and hexamethylenetetramine are responsible for its antimicrobial activity. Apart from antifungal activity, these compounds also possess antibacterial and antiviral activity and separately have been used for some applications [16, 17].

The main purpose of this study was to investigate antibacterial, antifungal and anti-phage properties of Petamcin-A. The obtained results could serve as a basis to develop its production for skin diseases treatment.

## Materials and Methods

### Microorganisms, growth conditions and antimicrobials used

Following microorganisms were used as test strains: bacteria *Escherichia coli* VKPM-M17 (Russian National Collection of Industrial Microorganisms at the Institute of Genetics and Selection of Industrial Microorganisms, Russia) (Laboratory control strain), *Pseudomonas aeruginosa* GRP3 (Soil and Water Research Institute, Iran) (Laboratory control strain), *Bacillus subtilis* WT-A1, isolated from metal polluted soils of Kajaran, Armenia, and *Bacillus licheniformis* WT (Department of Microbiology, Plants and Microbes Biotechnology, Yerevan State University (YSU), Armenia) (Laboratory control strains), *Micrococcus luteus* WT (Department of Microbiology, Plants and Microbes Biotechnology, YSU, Armenia) (Laboratory control strain), *Salmonella typhimurium* MDC 1754 (Microbial Depository Center, Armbiotechnology Scientific and Production Center, Armenia) (Laboratory control strain), *Staphylococcus aureus* MDC 5233 (Microbial Depository Center, Armbiotechnology Scientific and Production Center, Armenia) (Laboratory control strain), *Lactobacillus delbrueckii* subsp*. lactis* INRA-2010-4.2 (Department of Microbiology, Plants and Microbes Biotechnology, YSU, Armenia) (Probiotic strain), *Lactobacillus delbrueckii* subsp. *bulgaricus* INRA-2010-5.2 (Department of Microbiology, Plants and Microbes Biotechnology, YSU, Armenia) (Probiotic strain), *Escherichia coli* C-T4 (Eliava Institute of Bacteriophage*,* Microbiology and Virology, Georgia), yeasts *Candida albicans* WT-174 isolated from infected vaginal microbiota of hospitalized patients (Department of Botany and Mycology, YSU, Armenia) (Clinical strain), *Candida guilliermondii* HP-17 (Department of Botany and Mycology, YSU Armenia) (Laboratory control strain), *Debaryomyces hansenii* WT (French National Institute for Agricultural Research (INRA), France) (Laboratory control strain), mould *Mucor plumbeus* WT and *Geotrichum candidum* WT (INRA, France) (Food pathogens), *Penicillium aurantioviolaceum* WT, isolated from spoiled food, *Trichoderma viride* WT*,* isolated from spoiled food, and *Aspergillus flavus* WT isolated from spoiled food (Department of Botany and Mycology, YSU, Armenia) (Food pathogens).

LB medium was used for cultivation of bacteria (peptone 10 g l^−1^, yeast extract 5 g l^−1^, sodium chloride 10 g l^−1^, sucrose 5 g l^−1^, MgSO_4_ 0.5 g l^−1^), except *Lactobacillus delbrueckii* subsp*. lactis* and *Lactobacillus delbrueckii* subsp. *bulgaricus* which were cultivated in MRS (HiMedia, India). Beer wort agar (BWA) (Sigma-Aldrich, USA) and Sabouraud (glucose 40 g l^−1^, peptone 10 g l^−1^, yeast extract 5 g l^−1^) media were used for cultivation of fungi. To obtain solid medium, agar (9 g l^−1^) was added to nutrient media mentioned above.

12 % v/v acetic acid (one of the active compounds of Petamcin-A) as well as 30 μg ml^−1^ concentration of nystatin (“Borisov Plant of Medical Preparations” JSC, Borisov, Belarus) dissolved in 5 % DMSO and 0.2 % w/v chlorhexidine gluconate (“Arsanit” LLC, Yervan, Armenia) were used as control agents for comparative analysis of the Petamcin-A activity.

### Determination of antibacterial and anti-yeast activity

The bacteriostatic activity of Petamcin-A was tested by modified agar well diffusion assay [18]. 100 μl of overnight bacterial suspensions of indicator strains (adjusted to 10^6^ colony-forming unit (CFU) ml^−1^) were poured into Petri dishes. Subsequently, 25 ml medium was added on top of them at 50 °C and gently shaken. After medium solidification wells with a diameter of 8 mm were cut in it. 100 μl of compounds were added into wells. Plates were incubated at 37 °C except for *D. hansenii,* which was incubated at 27 °C. After 24 h zones of growth inhibition around the wells were measured.

For determination of minimal inhibitory concentrations (MIC) different concentrations of the preparation were tested by the method described above. The preparation was diluted in sterile distilled water. The maximal dilution which caused well defined growth inhibition zone of at least 2 mm was considered as MIC.

Bactericidal activity was determined by modified broth macrodilution method [19]. Approximately 1 mm^3^ parts of the medium from growth inhibition zones were cut off and subcultured in a liquid medium without antimicrobials. Tubes with media were incubated for 24 h. The growth of bacteria was estimated by measuring the optical density (OD) of culture media at 595 nm wavelength light using a spectrophotometer (GENESYS 10S UV–VIS, Thermo Scientific, USA). The absence of growth indicated about bactericidal activity of the preparation.

### Determination of anti-mould activity

Anti-mould activity of the preparation was explored by agar dilution assay [18]. BWA plates were inoculated with test strains and kept at 27 °C for 10 day for complete spore formation. Then spores were separated from mycelia by washing with saline solution and poured into tubes. The concentration of spores was calculated with hemocytometer. The preparations were poured into small (5 ml) Petri dishes in appropriate concentrations, then 2 ml of BWA medium at 50 °C was added and gently shaken. After solidification 10^4^ CFU ml^−1^ fungal spores were added. Petri dishes were incubated for 10 day at 27 °C. The absence of growth indicated about anti-mould activity. Different concentrations of preparations were used in order to determine the MICs.

### Determination of anti-phage activity

Anti-phage activity was determined by double agar overlay plaque assay [18]. Firstly, phage suspension was prepared. The concentration of plaque-forming units (PFU) was determined by the same method. In experimental group 30 μl of phage suspension at 10^11^ PFU ml^−1^ concentration and 30 μl of tested preparations were poured in a tube, while in the control group 30 μl saline solution was used instead of the preparations. The mixtures were incubated for 90 min at 37 °C. Then phage mixtures were diluted up to 10^−8^ in LB broth by serial ten-fold dilutions. *E. coli* C-T4 was pre-cultivated on slant agar then washed with LB broth and transferred into sterile tubes. The concentration of cells was determined measuring OD at 595 nm wavelength light. Appropriate dilution of phage (in volume of 1 ml) was poured into a tube. *E. coli* C-T4 (100 μl) was added and then 6 ml of LB containing 0.7 % agar at 50 °C was added on top. The mixture was shaken and poured in Petri dishes containing 20 ml 1.8 % LB agar. The plates were swirled, left to dry for 10 min at room temperature and incubated for 24 h at 37 °C. Viable phages form plaques on the seeded plates which could be enumerated. The number of viable phage particles in stock solution was determined by multiplying plaque numbers with dilution factor. The efficiency of the preparations was determined by comparing the amount of viable phage particles from experimental and control groups.

### Data processing

All experiments were independently repeated three times. Obtained data were processed; standard deviations and standard errors were calculated using GraphPad Prism 5.03 (GraphPad Software, Inc.; USA) software. *P*-values were determined by Student’s *t*-test with R 3.1.0 (The R foundation of statistical computing, Vienna, Austria*,* 2014) software.

## Results

Antimicrobial activity of Petamcin-A against different test strains was investigated. The obtained data showed that Petamcin-A has a high antibacterial activity against all tested strains (Table 1). Moreover, it was more effective compared with control agents. Petamcin-A was active against both prokaryotic and eukaryotic microorganisms. The preparation was more effective against tested gram-positive bacteria than gram-negative ones (see Table 1).

**Table 1:** Antibacterial and anti-yeast activity of Petamcin-A in comparison with 30 μg mlx^−1^ nystatin, 0.2 % chlorhexidine and 12 % acetic acid^a^

Test strains	Growth inhibition zones, mm			
	Petamcin-A	Chlorhexidine	Nystatin	Acetic acid
*Escherichia coli*	34 ± 0.9	21 ± 0.9	0	18 ± 0.5
*Pseudomonas aeruginosa*	36 ± 1	12 ± 0.7	0	25 ± 0.8
*Salmonella typhimurium*	44 ± 0.6	24 ± 1	0	31 ± 0.6
*Bacillus licheniformis*	54 ± 1.5	21 ± 0.6	0	35 ± 0.7
*Bacillus subtilis*	43 ± 1.2	19 ± 0.5	0	23 ± 0.6
*Lactobacillus delbrueckii* subsp. *lactis*	42 ± 1.2	23 ± 0.7	0	22 ± 0.9
*Lactobacillus delbrueckii* subsp. *bulgaricus*	41 ± 1	22 ± 1	0	21 ± 0.6
*Micrococcus luteus*	63 ± 1.5	31 ± 0.11	21 ± 0.9	45 ± 1
*Staphylococcus aureus*	56 ± 1	20 ± 0.8	0	29 ± 0.9
*Candida albicans*	31 ± 0.8	25 ± 0.7	27 ± 1	22 ± 0.4
*Candida guilliermondii*	45 ± 01.1	28 ± 0.9	26 ± 0.8	24 ± 1.1
*Debaryomyces hansenii*	79 ± 2	40 ± 1.2	32 ± 1.2	35 ± 0.1

^a^All experiments were independently repeated three times. Mean values with standard deviations are presented, *p* ≤ 0.003. For strains and details, see Materials and methods

MICs of Petamcin-A and 12 % acetic acid against *S. aureus* and *E. coli* were determined (Table 2). Petamcin-A maintained its activity against *S. aureus* till 14 μl ml^−1^ concentration*,* while its MIC for *E. coli* was 18 μl ml^−1^. MICs of 12 % acetic acid were higher-33 μl ml^−1^ and 50 μl ml^−1^ for *E. coli* and *S. aureus,* respectively.

**Table 2:** Determination of MICs of Petamcin-A and 12 % acetic acid against some test strains^a^

Strains	MIC, μl ml^−1^	
	Petamcin-A	Acetic acid
*S. aureus*	14	33
*E. coli*	18	50
*D. hansenii*	25	67
*T. viride*	15	25

^a^All experiments were independently repeated three times. Mean values with standard deviations are presented, *p* ≤ 0.0023. For details, see Materials and methods

The results demonstrated the bactericidal activity of Petamcin-A against almost all tested bacterial strains, with the exception of *B. subtilis* WT-A1, against which the activity was bacteriostatic. Moreover, Petamcin-A retained bactericidal activity against *S. aureus* till 33 μl ml^−1^ concentration (Table 3). In case of *E. coli*, bactericidal activity of the preparation was observed till 100 μl ml^−1^ concentration. Even though 12 % acetic acid showed bactericidal activity too, it lost this activity started from 200 μl ml^−1^ concentration. This could testify that in low concentrations of Petamcin-A its component acetic acid is not the main cause of its bactericidal studies showed that Petamcin-A had higher activity against all tested yeast strains compared with control agents. It demonstrated maximal activity against *D. hansenii* (see Table 1). Moreover, Petamcin-A had fungicidal effect against all tested yeast strains and lost this activity starting from 100 μl ml^−1^ concentration against *Candida* strains, and from 50 μl ml^−1^ concentration against *D. hansenii*. Chlorhexidine and nystatin also expressed fungicidal activity against tested yeast strains, whereas 12 % acetic acid showed only static activity against *C. albicans*, but was fungicidal against *C. guilliermondii* and *D. hansenii*.

**Table 3:** Determination of MBCs of Petamcin-A and 12 % acetic acid against some test strains^a^

Strains	MBC, μl ml^−1^	
	Petamcin-A	Acetic acid
*S. aureus*	33	67
*E. coli*	100	200
*C. albicans*	100	–
*C. guilliermondii*	100	Undiluted
*D. hansenii*	50	Undiluted

^a^All experiments were independently repeated three times. Mean values with standard deviations are presented, *p* ≤ 0.002. For details, see Materials and methods

The investigations of anti-mould activity showed that both Petamcin-A and acetic acid inhibited the germination of all tested mould spores till 50 μl ml^−1^ concentration. However, determination of MIC against *T. viride* showed that Petamcin-A inhibited spore germination even at 10 μl ml^−1^ concentration, whereas 12 % acetic acid MIC for *T. viride* spores was 20 μl ml^−1^.

**Fig. 1:**
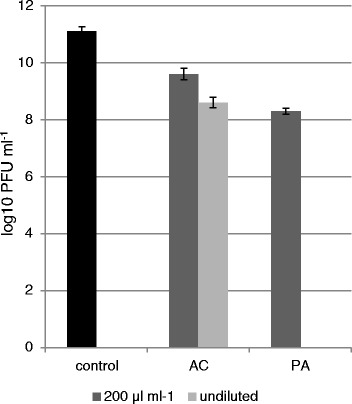
Anti-phage activity of Petamcin-A (PA) and 12 % acetic acid (AC) and their 200 μl ml^−1^ concentrations against T4 phage of *E. coli* C-T4 after 90 min exposure time. Saline solution was used as a negative control. Concentrations of PFU of T4 phage of *E. coli* C-T4 were illustrated with Log10 values. Mean values with standard deviations are presented, *p* ≤ 0.005. For details, see Materials and methods

In addition, Petamcin-A demonstrated high activity against T4 phage of *E. coli* C-T4 completely inhibiting its growth. 12 % acetic acid also expressed anti-phage activity, but with less efficiency (Fig. 1). It caused only 2.4 Log_10_ reduction of phage units. 200 μl ml^−1^ concentration of Petamcin-A also demonstrated considerable activity reducing phage numbers by 2.6 Log_10_, while the use of 100 μl ml^−1^ concentration of 12 % acetic acid resulted in only 1.4 Log_10_ reduction of *E. coli* C-T4.

## Discussion

Petamcin-A is patented as antifungal preparation [14] for the treatment of various fungal skin infections. Its active antimicrobial components are acetic acid and hexamethylentetramine which separately have been used in various medical applications, including the treatment of skin infections for a long time [16, 17, 20]. However, there is no data about their activity when applied together. Thus, it was very interesting to study the antibacterial and antiviral activity of Petamcin-A as a complex preparation.

Obtained data showed high efficiency of Petamcin-A against all tested strains, including bacteria, yeast, moulds and phages. Studies of antibacterial activity of the preparation illustrated that it was more effective than its component acetic acid and control agents. Moreover, its activity was higher against gram-positive bacteria in comparison with gram-negative ones. Such a difference was previously described for acetic acid [21]. At the same time there are no data indicating such differences for another active component of Petamcin-A–formaldehyde, which is released by decomposition of hexamethylenetetramine in even slightly acidic environment [22–24]. This allows speculating that this effect of Petamcin-A may be due to acetic acid.

The results allow to make some assumptions concerning Petamcin-A’s components activities. Particularly, it was determined, that in case of gram-negative bacteria growth inhibition zones by Petamcin-A were bigger than that by 12 % acetic acid (see Table 1). This difference was maximal (16 mm) for *E. coli*. Conversely, this difference in case of gram-positive bacteria was minimal (18 mm) for *M. luteus*, while it reached maximal value (27 mm) for *S. aureus.* Accordingly, it could be speculated that formaldehyde was more effective against gram-negative bacteria compared with gram-positive ones. Such a phenomenon was not described previously.

It is important to mention that Petamcin-A was particularly active against one of the most common causative agents of skin infections-*S. aureus* [25].

Petamcin-A killed all tested bacterial strains, except *B. subtilis* WT-A1. Bactericidal activity of the preparation is due to both acetic acid and formaldehyde. Many researchers reported about bactericidal activity of acetic acid and formaldehyde [13, 16, 17, 21, 26]. This means that both these components are responsible bactericidal activity of Petamcin-A. Two endospore forming bacteria were used in the study. Petamcin-A activity against *B. licheniformis* WT was bactericidal, whereas it was bacteriostatic against *B. subtilis* WT-A1. This may lead to speculations that there was a difference in the activity of Petamcin-A against sporulating and non-sporulating bacterial strains. However, *B. subtilis* WT-A1 strain was isolated from heavy metal polluted soils. Some studies state that genes responsible for heavy metal tolerance and antibiotic resistance are located in the same plasmid meaning there is a correlation between these two traits [27, 28]. Consequently, it can be assumed that *B. subtilis* WT-A1 strains have resistance against antimicrobials. Thus, the reason of Petamcin-A’s only bacteriostatic activity may be due to enhanced resistance of a particular strain, instead of its endospore forming ability. There is no evidence about acetic acid activity against bacterial spores, but many authors state about sporocidal activity of formaldehyde [21, 29, 30]. This affirms that Petamcin-A possesses activity against spores.

Petamcin-A showed high antifungal activity against tested three yeast and five mould strains. MIC determination of Petamcin-A and 12 % acetic acid against *T. viride* demonstrated that both active components of the preparation have their contribution in antifungal activity. Literature data confirms these results. For instance, high antifungal activity of acetic acid even at low concentrations against *Aspergilus flavus, Aspergilus luchuensis, Mucor spp., Penicillium oxalicum etc.* was shown [31]. High fungicidal activity of formaldehyde was also reported [29].

The investigation of anti-phage activity showed that there was a considerable difference between activity of Petamcin-A and its component acetic acid. This suggests that antiviral activity of Petamcin-A is only partially due to acetic acid and, therefore, formaldehyde also have noticeable implementation in this activity. According to literature data acetic acid has been reported to be less effective against viruses compared with formaldehyde which has confirmed our results [26, 29]. It was shown that 6 % acetic acid showed only 0.32 Log_10_ reduction of *Poliovirus* after 5 min exposure time [17].

The mechanisms of Petamcin-A’s antimicrobial activity can be derived based on activities of its two components-acetic acid and formaldehyde. On the other hand, it is possible that together they could gain some new advantageous properties. Consequently, some further investigations are needed to understand the mode of Petamcin-A action as a complex preparation.

It is known, that mechanisms that underlie the activity of formaldehyde are due to its reactions with proteins and nucleic acids [29, 32]. Particularly, it alkylates amino and sulfhydryl groups of proteins, as well as nitrogen atoms of the purine rings of nucleic acids [32]. Therefore, this component is active against almost all types of microorganisms.

The modes of action of another active component of Petamcin-A-acetic acid against microbes have not been understood completely yet. But it is assumed that mainly its undissociated forms are responsible for antimicrobial activity [33, 34]. They could passively cross the cell wall and dissociate at neutral pH within the cells. This brings to a decrease of internal pH and cause further inhibitory effects on microbes. For example, interior acidic pH causes denaturation of acid-sensitive proteins and DNA, consumption of energy during regulation of internal pH, *etc*. It has also been proposed that acetic acid brings to interruption of the action of bacterial proton pumps responsible for ion regulation [33, 34].

The main part of this work was done using laboratory control strains. It would be interesting to investigate Petamcin-A’s activity against more clinical isolates and human pathogenic strains. This will be the next step of the work which could help to broaden its potential applications.

## Concluding remarks

Petamcin-A has a high antimicrobial activity against all tested bacterial, yeast and mould strains. The preparation also exhibited high anti-phage activity. Furthermore, taking into account that Petamcin-A has not shown observable toxicity on skin and its components are not expensive, it can be advantageous for management of various skin medical conditions.

As far as Petamcin-A expressed antimicrobial activity against various groups of microorganisms, it also may have perspective to be used as a disinfectant but further investigations are needed.
